# Korean Model for Post-acute Comprehensive rehabilitation (KOMPACT): The Study Protocol for a Pragmatic Multicenter Randomized Controlled Study on Early Supported Discharge

**DOI:** 10.3389/fneur.2021.710640

**Published:** 2021-09-08

**Authors:** Won Kee Chang, Won-Seok Kim, Min Kyun Sohn, Sungju Jee, Yong-Il Shin, Sung-Hwa Ko, Minsu Ock, Hyun Joo Kim, Nam-Jong Paik

**Affiliations:** ^1^Department of Rehabilitation Medicine, Seoul National University Bundang Hospital, Seoul National University College of Medicine, Seongnam, South Korea; ^2^Department of Rehabilitation Medicine, Chungnam National University College of Medicine, Chugnam National University Hospital, Daejeon, South Korea; ^3^Department of Rehabilitation Medicine, Pusan National University School of Medicine, Pusan National University Yangsan Hospital, Pusan, South Korea; ^4^Department of Preventive Medicine, Ulsan University Hospital, University of Ulsan College of Medicine, Ulsan, South Korea; ^5^Department of Nursing Science, Shinsung University, Dangjin, South Korea

**Keywords:** stroke, transitional care, early supported discharge, randomized controlled trials, quality of life, Korea

## Abstract

**Introduction:** Early supported discharge (ESD) is a transitional care model aimed at facilitating post-acute stroke patients' discharge to home. Previous studies have demonstrated that ESD provides equivalent patient and caregiver outcomes with superior cost-effectiveness compared to conventional rehabilitation (CR). This study intends to examine the feasibility of ESD in Korea.

**Methods and Analysis:** This study is designed as a multicenter assessor-blinded, randomized controlled trial. Ninety post-acute stroke patients with mild to moderate disability (modified Rankin Scale 1–3) will be recruited from three university hospitals (30 patients per hospital) in Korea and allocated to either the ESD group or the CR group in a 1:1 ratio. Patients in the ESD group will receive individualized discharge planning and goal setting, a 4-week home-based rehabilitation program, and liaison service to community-based resources by a multidisciplinary team. Patients in the CR group will receive rehabilitation practices according to their current hospital policy.

**Outcomes:** The primary outcome is the Korean version of the modified Barthel Index, and the primary endpoint was post-onset 3 months. Clinical outcomes, patient/caregiver reported outcomes, and socioeconomic outcomes will be measured at baseline, 1 month after discharge, 2 months after discharge, and 3 months after onset.

**Discussion:** The efficacy and cost-effectiveness of ESD can vary according to the healthcare system and sociocultural aspects. To establish ESD as an alternative transitional care model for post-acute stroke patients in Korea, its feasibility needs to be examined in prior. This study will add evidence on the applicability of ESD in Korea.

**Ethical Considerations:** The study protocol was reviewed and approved by the Institutional Review Board of Seoul National University Bundang Hospital (IRB number B-2012/654-308). The study protocol was registered at ClinicalTrials.gov (Identifier NCT04720820). Disseminations will include submission to peer-reviewed journals and presentations at conferences.

## Introduction

Stroke is a significant global healthcare problem, accounting for the second largest disease burden (1) and major health service costs (2). The numbers of both post-stroke survivors and associated healthcare costs are expected to increase in the future (3), which will lead to a strong need for the establishment of an efficient and effective transitional stroke care model.

Early supported discharge (ESD) is an approach that aims to accelerate the home discharge of post-acute stroke patients by providing an equivalent level of rehabilitation services in the patient's home setting by a specialized multidisciplinary team (4, 5). A recent meta-analysis study has shown that a well-organized ESD can reduce long-term dependency and admission to institutional care of stroke patients as well as reduce the length of hospital stay (6). However, studies on ESD have mostly been conducted in European countries (7–12), Canada (13), and Australia (14), and the effectiveness of ESD can vary in non-European countries due to differences in medical systems and costs, allocation of rehabilitation resources, and cultural aspects (15). Thus, the applicability of the ESD model in Asian countries, namely Korea, remains unclear and requires further validation.

This study aims to examine the effectiveness and economic impact of ESD in post-acute stroke patients in Korea by comparing it with conventional rehabilitation (CR) through a multi-center assessor-blinded randomized controlled trial.

## Materials and Methods

### Study Design

**Figure 1:**
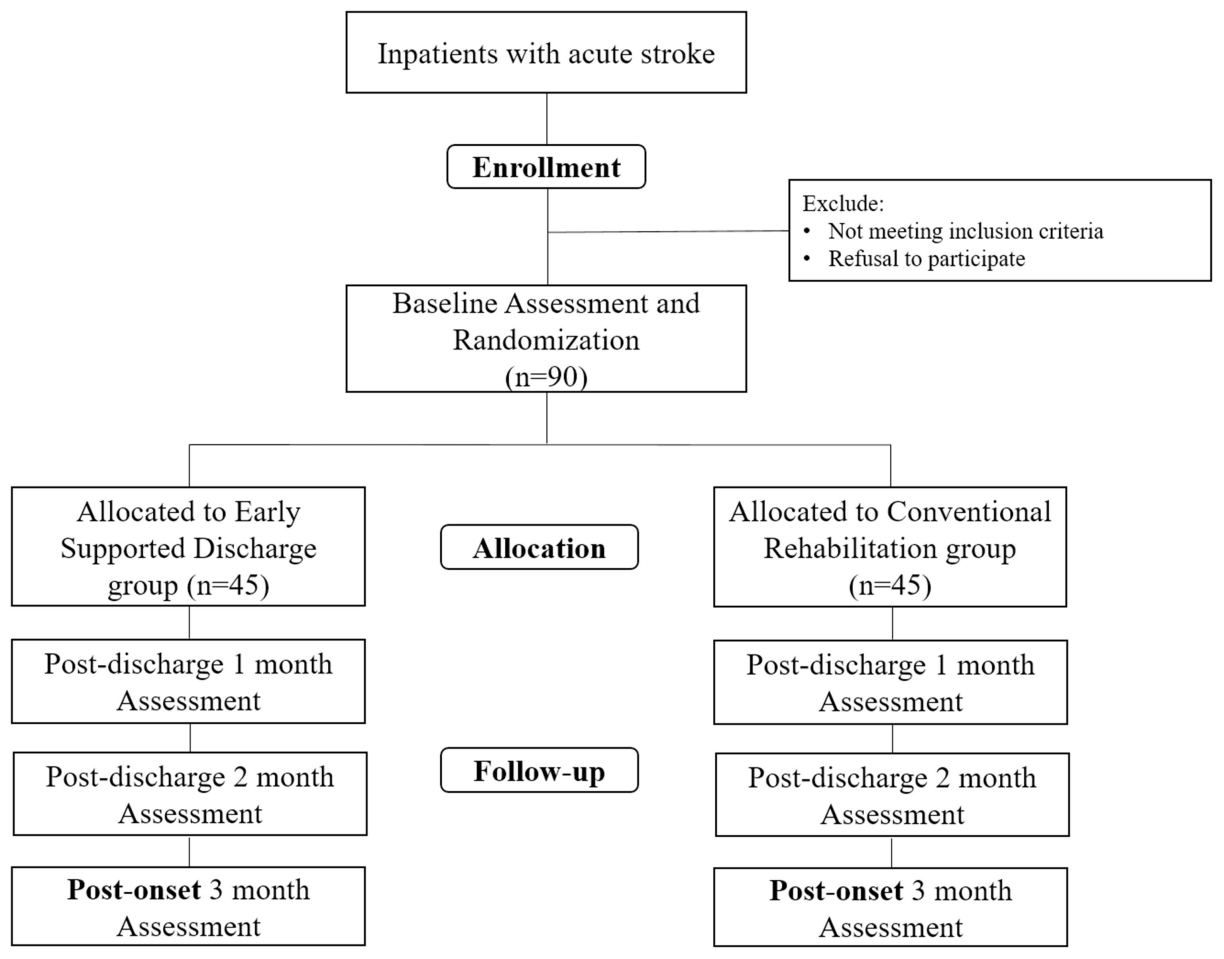
Flowchart of the study design.

This study is designed as a multi-center assessor-blinded, randomized controlled trial. Patients will be recruited from three national university hospitals in Korea: Seoul National University Bundang Hospital (Seongnam-si, Gyeonggi-do, Korea), Chungnam National University Hospital (Jung-gu, Daejeon, Korea), and Pusan National University Yangsan Hospital (Yangsan-si, Gyeongsangnam-do, Korea). Figure 1 shows an overall flowchart of the proposed study.

### Aim and Primary Hypothesis

This study aims to examine the effectiveness of ESD services in stroke patients with mild to moderate disability in Korea and establish it as an alternative to conventional rehabilitation services.

The primary hypothesis of the study is that the functional outcome of the patients in the ESD group would be non-inferior to those in the CR group, which was based on previous results of ESD trials (9, 13, 14). Producing equivalent functional outcomes with possibly reduced healthcare-related costs will prove that ESD is a feasible option for patients with stroke.

### Recruitment, Randomization, and Group Allocation

Patients will be screened for eligibility when referred to the rehabilitation department during acute stroke inpatient treatment. Eligible patients will be informed and enrolled by the study team. After the patient is enrolled, the patient will undergo baseline evaluation by the assessor and will be randomly allocated by the coordinator into either the ESD group or the CR group in a 1:1 ratio (total 30 patients per hospital). Randomization sequences were computer-generated using mixed block sizes of two and four, without stratification by the institution's biostatistician who is independent of the study team and will be managed by the coordinator in an opaque-sealed envelope at each study site. The patients' allocated group will be informed to the study team by the coordinator after the baseline evaluation is done. Patients' follow-up arrangements are managed by the study coordinator.

### Blinding

All evaluations (except for the ESD program satisfaction questionnaires) will be performed at each hospital by designated occupational therapist who is independent from the ESD team. The assessor will be blinded to the patients' intervention group throughout the study.

### Eligibility

This study aims to recruit acute stroke patients with mild to moderate disabilities who can be directly discharged to home after acute stroke treatment at the hospital. Major inclusion criteria are as follows: age > 20 years, planned to be discharged to home within 30 days from onset, initial modified Rankin Scale score 1–3, functional ambulation category score 3 or above, and no deficit of consciousness. Major exclusion criteria are as follows: transient ischemic attack, medically unstable, psycho-behavioral problems, or severe cognitive deficits that hinder participation in the ESD service. The detailed inclusion and exclusion criteria are listed in Table 1.

**Table 1:** Inclusion and exclusion criteria.

**Inclusion criteria**
**1. Patient who is admitted with clinical diagnosis of stroke 2. Patient who will be discharged to home within 30 days from onset 3. Patient who lives within 30-min distance from the discharged hospital with caregiver 4. Patient who's initial functional status is (measured within 14 days from onset) 1) Mild to moderate disability (mRS score 1–3) 2) Able to walk of transfer with on-man assist (FAC 3 or above) 3) No deficit of consciousness (K-NIHSS 1a, 1b, and 1c score 0)**
**Exclusion criteria**
**1. Patient who had TIA 2. Patient who is medically unstable 3. Patient who has indwelling catheter 4. Patient who's oral food intake is prohibited 5. Patient who has uncontrolled pain (with usage of NSAIDs or Opioids) 6. Patient who has psychobehavioral problems 7. Patient who has severe cognitive deficit (K-MMSE <15) 8. Patient who is unable to participate post-stroke rehabilitation program**

*mRS, modified Rankin Scale; K-MBI, Korean version of modified Barthel Index; FAC, Functional Ambulation Category; K-NIHSS, Korean version of the National Institute of Health Stroke Scale; TIA, Transient Ischemic Attack; NSAIDs, Non-Steroidal Anti-Inflammatory Drugs; K-MMSE, Korean version of the Mini-Mental State Exam*.

### Interventions

The ESD team at each hospital is comprised of a rehabilitation physiatrist, a physiotherapist, an occupational therapist, and a social worker (optional) who have experience in stroke patient care. The ESD service consists of individualized discharge planning and goal setting, 4-weeks of home-based tailor-made rehabilitation program, including at least 30 min of visiting physical therapy (PT) and occupational therapy (OT) sessions each at the patients' homes per week, additional PT, OT, and speech therapy (ST) at the hospital outpatient settings if needed, and liaison service to community-based resources as required. The team will discuss each patient's ESD program biweekly and modify the ESD program, if necessary. ESD programs will be set to meet patients' needs, with emphasis on adjusting to community living (16).

Patients in the CR group will receive current practices on post-acute stroke patients regarding discharge planning and follow-up rehabilitation services. Patients will receive outpatient-based PT, OT, and ST after discharge, if needed. Referral to community-based resources will be done according to the current hospital policy.

### Safety of the Health Professionals and the Patients

To secure the safety of health professionals during home rehabilitation services, ESD team will visit the patients' home in team of two or more people. Also the patients' caregiver will be mandatory to stay at home during the visit. ESD services will be terminated if there is significant risk to the team regarding safety. To minimize the risk of COVID-19 infection, ESD team will use personal protective equipment such as gloves and masks during the home rehabilitation services. The ESD services will be provided in accordance with the Korean government and the hospital's infection controlpolicies.

### Measures

There are several types of outcome measures in this study: clinical outcomes, patient and caregiver reported outcomes, and socioeconomic outcomes. Measures will be evaluated at baseline, 1 month after discharge, 2 months after discharge (if necessary), and 3 months after onset. Since the length of hospital stay is expected to be different in both groups (6), we have set the primary endpoint as 3 months after onset. The detailed outcome measures and evaluation timetable are shown in Table 2.

**Table 2:** Timetable and measures to be made.

**Measures**	**Screening**	**Baseline**	**1 months**	**2months^**+**^**	**3 months**
**Primary outcome**					
modified Barthel Index (K-MBI)		√	√		√
**Secondary outcomes—clinical**					
modified Rankin Scale (mRS)	√	√	√		√
Instrumental Activities of Daily Living (K-IADL)			√		√
Fall experience			√		√
Readmission rate			√		√
Mortality rate			√		√
**Secondary outcomes—patient reported outcomes**					
Patient Health Questionnaire-9 (PHQ-9)		√	√		√
Reintegration to Normal Living Index (K-RNLI)			√		√
Stroke Impact Scale 3.0 (K-SIS)			√		√
EuroQoL-5D-5L (EQ-5D-5L)		√	√		√
Zarit Burden Interview (K-ZBI 22)			√		√
ESD Program satisfaction questionnaires^*^			√		√
**Secondary outcomes—socioeconomic**					
Length of Hospital stay			√		
Direct cost (Hospital cost, Home-based rehabilitation cost)			√	√	√
Indirect cost (Transportation cost, Caregiver cost)			√	√	√
Productivity loss					√
**Other measures**					
Demographic/Social information	√	√			
Medical History	√	√			
ESD program participation rate	√				

* *Early Supported Discharge group only.*

+ *Measurement can be skipped if the time interval between 2 months after discharge and 3 months after onset is <2 weeks*.

### Primary Outcome

The primary outcome of this study is the Korean version of the Modified Barthel Index (K-MBI) (17). The K-MBI is a scale consisting of 10 items that measure the ability to perform activities of daily living (ADL). The K-MBI is widely used to assess the degree of function in stroke patients, and the score ranges from 0 to 100, with higher scores indicating better function of the patient.

### Secondary Outcomes—Clinical Outcomes

Clinical secondary outcomes include the modified Rankin Scale, Korean version of Instrumental Activities of Daily Living, fall experience, readmission, and mortality rates.

*The modified Rankin Scale (mRS)* is a clinician-reported ordinal scale that measures global disability. mRS is widely used to evaluate the gross functional outcomes of stroke patients (18). mRS ranges from 0 to 6 with 0 indicating no symptoms at all and 6 indicating death.

*The Korean version of Instrumental Activities of Daily Living (K-IADL)* measures a person's ability to perform activities that are required to live independently in the community (19). The K-IADL consists of 11 items (shopping, mode of transportation, ability to handle finances, housekeeping, preparing food, ability to use a telephone, responsibility for own medication, recent memory, hobbies, watching television, and fixing around the house). K-IADL ranges from 0 to 33, with lowerr scores indicating better ability.

*Fall experience, readmission, and mortality rates* are outcomes that are related to the safety and long-term dependency of community-dwelling post-stroke patients. Patients will be asked whether they had experienced a fall or serious fall-related injury, which is defined as an injury that requires medical treatment as a consequence of a fall (20). Readmission and mortality rates will be monitored by questioning the patient or caregiver at each follow-up schedule.

### Secondary Outcomes—Patient/Caregiver Reported Outcomes

*The Patient Health Questionnaire-9 (PHQ-9)* is a brief 9-item depression module used for screening depression (21). The PHQ-9 score ranges from 0 to 27, with higher scores indicating more severe depressive symptoms.

*The Korean version of the Reintegration to Normal Living Index (K-RNLI)* assesses the consequences of disease on the restoration of normal life with 11 domains scored using a visual analog scale (22).

*The Korean version of the Stroke Impact Scale 3.0 (K-SIS)* is used to identify patients' needs in multiple dimensions regarding health-related quality of life (HRQoL) (23). The scale consists of nine domains with each domain score ranging from 0 to 100. Higher scores indicate a better quality of life.

*EuroQoL-5D-5L (EQ-5D-5L)* is a globally used self-reported health status measure with five dimensions: mobility, self-care, usual activities, pain/discomfort, and anxiety/depression. Each dimension has five levels: no problems, slight problems, moderate problems, severe problems, and extreme problems.

*The Korean version of the Zarit Burden Interview (K-ZBI 22)* is the most widely used instrument for measuring caregiver burden (24). This scale contains 22-items and has 5 subscales assessing the subjective burden of caregivers: general strain, isolation, disappointment, emotional involvement, and environment. All items are scored on a scale from 1 to 4, with higher scores indicating a higher caregiver burden.

### Secondary Outcomes—Socioeconomic

*The length of hospital stay* will be obtained from electronic medical records (EMR). Both the total number of bed days and the number of days after randomization will be documented.

*Direct cost* includes the cost of hospital admission, outpatient clinic visits, and rehabilitation therapy (both home-based and outpatient-based). The cost generated at the study-site hospital will be acquired from EMR, while costs occurring at other hospitals or clinics will be obtained using a patient questionnaire. *Indirect cost* consists of transportation cost for visiting a hospital or clinic, caregiver cost (if a caregiver is employed to help the patient), and cost of modifying home to assist ADL (for example, installing a handrail on the wall to assist transfer). Indirect costs will be obtained using patient questionnaires.

*Productivity loss* will be calculated based on the human capital approach (25) determined by calculating the average monthly working hours and the average monthly wage obtained with the patient questionnaire.

### Data Collection and Management

Data from all patients will be collected by research team members and entered into iCReaT (http://icreat.nih.go.kr), a web-based clinical research and trial database management program developed by the Korea Centers for Disease Control and Prevention. The coordinating investigator will have access to the final study dataset, and the anonymized dataset will be available upon reasonable request to the corresponding author.

### Sample Size Estimation

The sample size of this study was estimated based on our primary hypothesis. The non-inferiority margin was defined as 50% of the changes in MBI scores from baseline to 3 months after onset from a previous ESD trial (14). With a non-inferiority margin of 6.2 and standard deviation of 10.6, the minimum sample size determined using PASS 2020 (NCSS, LLC. Kaysville, Utah, USA) was 37 patients in each group after applying a 5% probability of type 1 error and 80% statistical power. Allowing 18% of attrition per group, a total of 90 patients will be required for the study.

### Statistical Analysis

For the statistical analysis, the Student *t*-test or Mann-Whitney *U*-test will be used to analyze numeric variables according to the parametricity of the distribution. Chi-squire test will be used to analyze categorical variables. Subgroup analysis based on the baseline mRS scores (1–3) will be performed if there are sufficient participants in all three subgroups. For primary analysis, both the intent-to-treat (ITT) approach and per-protocol analysis approach will be applied. If the dropout rate exceeds 20% of the total patients in the final stage, ITT-based analysis will be performed after multiple imputations of missing data. *P* < 0.05 will be considered as statistically significant. The data will be analyzed using R software (version 4.0.2; R Foundation for Statistical Computing, Vienna, Austria).

## Discussion

ESD is currently considered an acceptable, cost-effective transitional care model for post-acute stroke patients in Europe, Canada, and Australia (6). However, the feasibility of ESD needs to be examined in the healthcare system environment of Korea, since the applicability of ESD depends on the healthcare system, stroke-related cost, and cultural aspect of the country it is implemented (15).

The healthcare system of Korea offers inpatient medical services at a relatively low cost with good accessibility compared to other developed countries (26) thus, the ESD program might not be an attractive option for stroke patients with moderate to severe disabilities. Therefore, we chose the target population of stroke patients with mild to moderate disabilities, which has been proven to have benefitted from ESD service (16). Another aspect that needs to be mentioned is the cultural perception of caregiving in Korea. It has been speculated that filial piety and familism of Asian culture have a positive effect on reducing caregiving burden (27), which can be advantageous in reducing the potential risk of increased caregiver burden caused by ESD (14).

The primary hypothesis of this study was set to test the non-inferiority of functional outcomes in patients undergoing ESD, compared with CR. This was based on previous studies (9, 12–14), and the results showed no significant difference in functional outcomes between the groups. Moreover, considering that our target population is post-acute stroke patients with mild to moderate disabilities, we expect the ability to perform ADL at our primary endpoint, 3 months after onset, will not be significantly different in both groups.

Current post-acute stroke rehabilitation in Korea is highly dependent on inpatient rehabilitation, resulting in a long hospital stay for stroke patients (28, 29). Since the economic burden of stroke in Korea is increasing (30) with productivity loss accounting for the largest portion (31), a search for a new model of post-acute stroke transitional care that could facilitate patients' reintegration to the community is essential.

In this study, we plan to investigate the impact of ESD on various dimensions; not only the functional outcomes and economic efficiency but also the social and cultural aspects. Sociocultural aspect is an important factor when implementing ESD into existing stroke care system (32). By assessing the impact of ESD in various aspects, our results will provide comprehensive information on its applicability.

In conclusion, this study aimed to examine the feasibility of ESD in post-acute patients with mild to moderate disability by comparing its efficacy and socioeconomic impact with the current rehabilitation system. This study provides evidence on the applicability of ESD in Korea.

## Ethics Statement

The study protocol was reviewed and approved by the Institutional Review Board of Seoul National University Bundang Hospital (IRB number B-2012/654-308). The study protocol was registered at ClinicalTrials.gov (Identifier NCT04720820). Written informed consent to participate in this study will be provided by the patients and/or their legal guardians.

## Author Contributions

WC, W-SK, MS, SJ, Y-IS, S-HK, MO, HK, and N-JP conceived the study and designed the study protocol. The draft of the manuscript was written by WC and revised by W-SK, MS, SJ, Y-IS, S-HK, and N-JP. All authors contributed to the manuscript and approved the submitted version.

## Funding

This work was supported by the Research Program funded by the Korea National Institute of Health (2020ER630601).

## Conflict of Interest

The authors declare that the research was conducted in the absence of any commercial or financial relationships that could be construed as a potential conflict of interest.

## Publisher's Note

All claims expressed in this article are solely those of the authors and do not necessarily represent those of their affiliated organizations, or those of the publisher, the editors and the reviewers. Any product that may be evaluated in this article, or claim that may be made by its manufacturer, is not guaranteed or endorsed by the publisher.
